# Soil Microbial Community Response Differently to the Frequency and Strength of Freeze–Thaw Events in a *Larix gmelinii* Forest in the Daxing’an Mountains, China

**DOI:** 10.3389/fmicb.2020.01164

**Published:** 2020-06-03

**Authors:** Minghui Liu, Fujuan Feng, Tijiu Cai, Shijie Tang

**Affiliations:** ^1^College of Life Science, Northeast Forestry University, Harbin, China; ^2^Key Laboratory of Sustainable Forest Ecosystem Management-Ministry of Education, Northeast Forestry University, Harbin, China; ^3^College of Forestry, Northeast Forestry University, Harbin, China

**Keywords:** freeze–thaw cycle frequency, freeze–thaw temperature fluctuation, soil microbial biomass, microbial community structure, enzyme activity

## Abstract

Sustained climate warming increases the frequency and strength of soil freeze–thaw (FT) events, which strongly affect the properties of soil microbial communities. To explore the responses and mechanisms of the frequency and strength of freeze–thaw events on soil microbial communities, a lab-scale FT test was conducted on forest soil in permafrost region from the Daxing’an Mountains, China. The number of FT cycles (FTN) had a greater effect on microbial communities than FT temperature fluctuation (FTF). The FTN and FTF explained 20.9 and 10.8% of the variation in microbial community structure, respectively, and 22.9 and 11.6% of the variation in enzyme activities, respectively. The total and subgroup microbial biomass, the ratio of fungi to bacteria (F/B), and C- and N-hydrolyzing enzyme activities all decreased with an increase in FTN. Among microbial groups, arbuscular mycorrhizal fungi (AMF) were the most sensitive to FT events. Based on the changes of F/B and AMF, the reduction in soil carbon sequestration caused by frequent FT events can be explained from a perspective of microorganisms. Based on redundancy analysis and Mental Test, soil moisture, total organic carbon, and total nitrogen were the major factors affecting microorganisms in FT events. In the forest ecosystem, soil water and fertilizer were important factors to resist the damage of FT to microorganism, and sufficient water and fertilizer can lighten the damage of FT events to microorganisms. As a result of this study, the understanding of the responses of soil microorganisms to the variation in FT patterns caused by climate changes has increased, which will lead to better predictions of the effects of likely climate change on soil microorganisms.

## Introduction

In 2019, a paper published in *Nature Reviews Microbiology* warned humanity that the effects of climate change depend heavily on the responses of microorganisms (Cavicchioli et al., 2019). However, the responses of microorganisms are rarely the focus of studies on climate change, which fundamentally limits understanding of the biosphere and its response to climate change. A later report further emphasized that the ecosystem services provided by soil microorganisms would be vital to establish and maintain ecosystem stability under future climate change (Jansson and Hofmockel, 2020). Thus, there is an urgent need to understand the repercussions of climate change on soil microorganisms and the biogeochemical processes that they drive. Increased understanding of the microbial traits that confer ecosystem resilience to climate change will lead to better predictions and management of ecosystem responses.

The continuous climate warming has reduced the snow cover in the Northern Hemisphere (Sorensen et al., 2018). Snow keeps the soil warmer and reduces soil temperature variability (Wang et al., 2003). With the loss of the insulation provided by snow cover, minimum soil temperatures decrease, and the frequency and severity of freeze–thaw (FT) cycles increase (Sulkava and Huhta, 2003; Groffman et al., 2011; Tan et al., 2014; Boswell et al., 2020). Any perturbation in FT cycles can strongly influence soil microbial community structure and function (Schimel et al., 2007; Haei et al., 2011), and changes in microbial communities lead to changes in soil biochemical processes (Tan et al., 2014; Cheng et al., 2018; Xiao et al., 2019), which can further adversely affect the change in climate (Cavicchioli et al., 2019). Field observations and laboratory simulations are common methods to study the effects of FT events on microbial communities. In laboratory simulations, the patterns of the FT process can be better controlled, providing detailed insight into the effects of the FT process on microorganisms (Song et al., 2017). However, a consensus has not been reached on whether the effects of FT patterns on soil microbial biomass and activities are positive or negative or on the sensitivity of subgroups of microorganisms to FT events (Haei et al., 2011; Zhang et al., 2017). In addition, most studies focus only on how the number of FT cycles (FTN) or the FT temperature fluctuation (FTF) affects the microbial community, whereas the study of specific combinations of these factors often helps to increase understanding of the mechanisms of change (Rillig et al., 2019). Thus, studies on the effects of both FTN and FTF on soil microbial communities are needed. The approach used in this study will help to further understand the feedbacks of soil microorganisms to current climate changes. In addition, the study provides a theoretical framework to address microorganisms strategies aiming to counteract the negative environmental effects of FT cycles.

The Daxing’an Mountains are at the southern edge of the permafrost region in Eurasia with a large permafrost area and seasonally frozen soil. The continuous changes in northern ecosystems are considered as the forerunners of ecological change in the circum-Arctic area under climate warming (Li et al., 2020). The annual temperature in this area has been increasing at the rate of 0.32°C per decade (*P* < 0.01; Supplementary Figure S1) in the past 60 years because of climate change. The increase is higher than that of the global average increase of 0.12 [0.08 to 0.14] °C per decade since 1951 (1951–2012) as published by the Intergovernmental Panel on Climate Change in 2014 (IPCC, 2014), which indicates that the Daxing’an Mountains experience more extreme temperature fluctuations and more frequent FT events. *Larix gmelinii*, the zonal vegetation in the Daxing’an area, is an important part of the coniferous forests in northern Eurasia and represents a coniferous forest in the cold temperate zone, accounting for more than 70% of forests in the Daxing’an Mountains (Wang et al., 2001). *L. gmelinii* also plays an important role in keeping the ecological balance of the Daxing’an Mountains.

How the soil microbial community responds to changes in the pattern of FT events in the Daxing’an Mountains remains unclear. Therefore, the following hypotheses were proposed to better understand the response of the microbial community: (1) FTN and FTF will have different effects on the activity and structure of soil microbial communities, and (2) the FT process will lead to a decline in biomass and activities of soil microbial communities. To test these hypotheses, laboratory simulations were used to investigate the effects of FT patterns on properties of soil microbial communities (at depths of 0–10 and 10–20 cm) in a *L. gmelinii* forest in the permafrost region of the northern Daxing’an Mountains. The soil respiration quotient (qCO_2_) and enzyme activities were used as indicators of microbial activity, and phospholipid fatty acids (PLFAs) were used as indicators of microbial community structure and biomass. To further address the first hypothesis, the effects of FTN and FTF on soil characteristics related to soil microbial metabolism and growth in previous studies were reviewed and compared with the results of this study. The aims of this study were (1) to compare the effects of FTN and FTF on the biomass, structure, and activity of soil microbial communities; (2) to explore how FTN and FTF change the biomass, structure, and activity of soil microbial communities; and (3) to identify the potential drivers of changes in microbial community properties during the FT process. The results will reveal the patterns of responses of soil microorganisms to the changes in FT patterns caused by climate warming, in addition to providing practical data and a theoretical reference for methods that use soil microorganisms to mitigate the negative effects of climate change.

## Materials and Methods

### Site Description

The study was conducted at the Heilongjiang Mohe Forest Ecosystem Research Station in the permafrost region of the northern Daxing’an Mountains of Northeast China. The area has a typical cold temperate continental monsoon climate with a mean annual air temperature of −4.3°C. The mean annual precipitation ranges from 430 to 550 mm and is highest in July and August. The frost-free period is 85–110 days. The mean annual temperature varies from −11.81 to 4.31°C, and the annual sunshine is approximately 2,427 h. The mean annual relative humidity is approximately 70%. The soil is predominantly brown coniferous forest soil. The zonal vegetation is a coniferous forest with *L. gmelinii* as the dominant tree species. The study site was a permanent 1-ha plot of *L. gmelinii* forest at the research station (53°28′03′′ N, 122°20′23′′ E). The plot is at an altitude of 297 m. In the plot, the average diameter at breast height was 10.23 cm, the average tree height was approximately 13.5 m, and the canopy density was 0.75. The density of *L. gmelinii* was 2,030 ha^–1^. The associated tree species were *Betula platyphylla*, *Pinus sylvestris* var. *mongolica*, and *Populus davidiana*, and the shrubs were *Ledum palustre* var. *dilatatum*, *Rhododendron dauricum*, *Vaccinium uliginosum*, and *V. vitis-idaea*, among others.

### Soil Collection and Experimental Design

In October 2018, three quadrats (20 m × 20 m, with the two nearest quadrats separated by approximately 20 m) were set up in the *L. gmelinii* forest. The litter was removed, and in each quadrat, 48 soil cores (7.5-cm diameter and 30-cm depth) were collected, avoiding physical disturbances of the soil structure (Hentschel et al., 2008). They were refrigerated and taken immediately to the laboratory. The sides and bottoms of the soil cores were wrapped with 3.5-cm-thick polyethylene and polyurethane as insulating material for FT treatment. This insulation was used to make heat interchange only occur at the soil surface, thus better mimicking the natural environmental condition. The soil cores were divided into three FT temperature treatments: (1) constant 4°C (C); (2) FT cycles between −4 and 4°C (H); and (3) FT cycles between −14 and 4°C (L). After constant culture at 4°C for 7 days, to simulate the process of soil FT in the study area as much as possible, the soil freezing and thawing were controlled by slowly decreasing and increasing the temperature in a temperature chamber. The temperature was slowly reduced to −4 or −14°C for 24 h and then slowly increased to 4°C for 24 h, which was regarded as a freeze–thaw cycle (Supplementary Figure S2). Three replicate soil columns per temperature treatment were collected after the completion of 1, 3, 7, or 12 FT cycles. From the same temperature treatment and the same FT cycle, the 0–10 and 10–20 cm layers of the soil columns were separately sieved through a 2-mm mesh to eliminate plant residues and roots than homogenized. Approximately 100 g of each soil sample was air dried to determine the soil physicochemical properties and enzyme activities. The remaining fresh soil samples were used immediately to determine microbial biomass carbon (MBC), PLFA content, and soil respiration.

### Soil Physicochemical Properties

Soil water content (SWC) was determined gravimetrically by oven-drying the soil for 8 h at 105°C. The soil pH was measured in a 1:2.5 (w/v) soil:water suspension solution by a pH meter (PHS-3C, INESA Scientific Instrument Company, China). Total nitrogen (TN) and total organic carbon (TOC) were measured in soil samples by using an EuroEA3000 element analyzer (Leeman Company, United States). C/N ratio was calculated by the ratio of TOC and TN. To determine dissolved organic carbon (DOC), soil samples were extracted by a 0.5 mol L^–1^ K_2_SO_4_ solution at 1:2.5 (w/v) soil:water. The suspension was shaken at 280 rpm for 30 min, centrifuged at 4,000 rpm for 30 min, and then filtered through a 0.45 μm filter. The carbon content of the extracted solution was determined with an Elementar Vario Max element analyzer (Elementar, Germany). NH${}_{4}^{+}$-N and NO${}_{3}^{-}$-N in soil samples were extracted with 2M of KCl and analyzed using a Continuous-Flow AutoAnalyzer (AA3, Bran+ Luebbe, Norderstedt, Germany).

### Microbial Biomass Carbon and Soil Enzyme Activity

MBC was estimated using the fumigation–extraction method (Vance et al., 1987). In brief, fresh soil samples were fumigated with ethanol-free chloroform for 24 h in the dark at 25°C. Fumigated soil was then extracted with a K_2_SO_4_ solution; non-fumigated soil was treated the same. MBC was determined using an Elementar Vario Max element analyzer (Elementar, Germany) and according to the difference in C content between fumigated and non-fumigated soils with an internal conversion coefficient of 0.45.

Carbon (C)-hydrolyzing enzyme activity [β-glucosidase (BG) and β-cellobiosidase (CBH)] and Nitrogen (N)-hydrolyzing enzyme activity [*N*-acetyl-glucosaminidase (NAG) and L-leucine aminopeptidase (LAP)] were measured by a fluorimetric microplate method (Marx et al., 2001). For each enzyme assay, the enzyme substrate, Enzyme Commission number, abbreviation, and enzyme function are shown in Table 1. The standard used for the enzyme assays was 4-methylumbelliferone or 7-amino-4-methylcoumarin. In brief, 1 g of soil sample was mixed with 125 mL of 50 mmol⋅L^–1^ sodium acetate buffer (pH = 5.4, similar to the soil sample pH) by magnetic stirring for 1 min. During shaking, 50 μL of 200 μmol L^–1^ substrate and 200 μL of soil suspension were dispensed in wells of 96-well black microplates as a sample assay (four analytical replicates each). Similarly prepared were the blank (50 μL of buffer + 200 μL of suspension), negative control (50 μL of substrate + 200 μL of buffer), standard control (50 μL of standard + 200 μL of suspension), and reference standard (50 μL of standard + 200 μL of buffer). Microplates were incubated for 4.0 h at 20°C, and the reactions were stopped using 10 μL of 1 mol⋅L^–1^ NaOH. The released fluorescence was analyzed using a multifunctional fluorimetric plate reader (Tecan Infinite 200 PRO, TECAN Group, Ltd., Männedorf, Switzerland) at 365 nm excitation and 450 nm emission. Enzyme activities are expressed as absolute enzyme activity, which was obtained by normalizing by MBC as nmol fluorescence⋅h^–1^⋅g^–1^ MBC.

**TABLE 1:** Substrate, enzyme commission number (E.C.), abbreviation, and function of enzymes in soil assays.

**Enzyme**	**Substrate**	**E.C.**	**Abbreviation**	**Function**
Alkaline phosphatase	4-Methylumbellifery-phosphate	3.1.3.1	AP	Mineralizes phosphate groups from soil organic matter
β-Glucosidase	4-MUB-β-d-glucoside	3.2.1.21	BG	Releases glucose from cellulose
β-Cellobiosidase	4-MUB-β-d-cellobioside	3.2.1.91	CBH	Releases disaccharides from cellulose
*N*-Acetyl-glucosaminidase	4-MUB-*N*-acetyl-β-d-glucosaminide	3.2.1.30	NAG	Cleaves the amino sugar *N*-acetyl-β-d-galactosamine from chitin
l-Leucine aminopeptidase	l-Leucine-AMC	3.4.11.1	LAP	Degrades protein into amino acids

*MUB, 4-methylumbelliferone; AMC, 7-amino-4-methylcoumarin.*

### Soil Respiration Quotient

The soil respiration rate was assessed by trapping respired CO_2_ in NaOH (Guo et al., 2017). Dry-equivalent soil samples, 15 g, were adjusted to 60% of water-holding capacity and placed in 500 mL plastic jars to pre-incubate at 25°C for 8 h. Then, a plastic bottle containing 5 mL of 0.1 mol⋅L^–1^ NaOH solution was put into each jar to capture CO_2_ at 25°C for 24 h. Controls without soil were treated the same. Four replications were prepared for each group. The amount of CO_2_ evolved from the incubated soil was determined via titration against 0.05 mol⋅L^–1^ HCl. The soil metabolic quotient (qCO_2_) was calculated as the ratio of soil respiration rate and MBC (Anderson and Domsch, 1990).

### Soil Phospholipid Fatty Acids

Soil microbial community structure was analyzed using PLFAs as described previously (Bossio and Scow, 1998). In brief, 8 g of fresh soil was extracted with a liquid mixture of chloroform:methanol:phosphate buffer (1:2:0.8 by volume) for 2 h. After centrifugation, the supernatant was transferred to a separatory funnel and mixed with 12 mL of chloroform and 12 mL of phosphate buffer for 2 min. The lower phase was then collected and extracted with 23 mL of the liquid mixture for 30 min. After centrifugation, the separated mixture was allowed to set overnight. The lower phase was concentrated under N_2_ at a temperature of 30–32°C and then used in lipid fractionation. The concentrated lipid extract was dissolved in 200 μL of chloroform, and lipids were fractionated on solid phase extraction columns (Supelco, Inc., Bellefonte, PA, United States). Neutral and glycol lipids were eluted by 5 mL of chloroform and 10 mL of acetone; polar lipids were then eluted by 5 mL of methanol. The methanol phase was then collected and dried under N_2_ at a temperature of 30–32°C. The dried lipids were mixed with 1 mL of a 1:1 solution of methanol and toluene and 1 mL of 0.2 mol⋅L^–1^ potassium hydroxide, heated to 37°C for 15 min, and then after extraction with n-hexane dried under N_2_. Finally, fatty acid methyl esters were detected by gas chromatography-mass spectrometry (Agilent 6850 series gas chromatograph, United States) using an HP-5 capillary column (25.0 m × 200 mm × 0.33 mm) with N_2_ as the carrier gas. The concentrations of each PLFA were calculated based on 19:0 internal standard concentrations and biomass is expressed as nmol per gram dry soil. The PLFAs used as biomarkers are shown in Table 2. PLFAs not assigned as biomarkers were included in total PLFA (tPLFA) yields.

**TABLE 2:** Phospholipid fatty acids (PLFAs) used as biomarkers.

**Microbial group**	**Fatty acid type**	**Phospholipids fatty acid signatures**	**References**
Gram-positive bacteria	a-/I-Branched fatty acids	12:0 iso, 13:0 iso, 13:0 anteiso, 14:0 iso, 15:0 iso, 15:0 anteiso, 16:0 iso, 16:0 anteiso, 17:0 iso, 17:0 anteiso, 18:0 iso, 19:0 iso, 19:0 anteiso, 20:0 iso	Bossio et al., 2006; Yao et al., 2018
Gram-negative bacteria	Monounsaturated fatty acids and cyclopropane fatty acids	11:0; 10:0 2OH, 2OH 12:0; 3OH i14:0;14:1 w5c, 15:1 w5c, 15:1 w6c, 16:1 w9c, 16:1 w7c, 16:0 2OH, 17:1 w8c, 17:1 w7c, 18:1 w7c, 18:1 w5c, 20:1 w9c,	Kourtev et al., 2002; Dong et al., 2014; Yao et al., 2018
		3OH 12:0; 2OH 13:0; 2OH 17:0; 2OH 18:0;3OH 18:0; 3OH i11:0; 3OH i12:0; 3OH i15:0; 3OH i16:0; 3OH i17:0; 2OH 16:1; 2OH 18:1, 12:0; 14:0, 15:0, 17:0,	
Actinomycetes	Methyl branched fatty acids	16:0 10-methyl, 17:0 10-methyl, 18:0 10-methyl	Bossio et al., 2006
Fungi		18:2 w6c, 18:2 w9c	Bååth and Anderson, 2003; Zhang et al., 2018
AM Fungi		16:1 w5c	Kaiser et al., 2010

### Statistical Analyses

One-way ANOVA was used to determine the effects of FT patterns on soil enzyme activity and subgroup microbial biomass. Before conducting ANOVA, the normality and homoscedasticity of the residues were verified by the Kolmogorov–Smirnov test and Levene’s test, respectively. *Post hoc* analyses were conducted using least-significant difference tests. The significance level was set at *P* < 0.05. Repeated measures ANOVA was used to analyze the effects of FTN, FTF, soil layers, and their interactions on microbial community composition and activity. Multivariate regression tree (MRT) analysis was used to evaluate the correlations between FTN, FTF, and soil layer and soil microbial community structure (De’ath, 2002) and was conducted using the package ‘mvpart’ in the software R (v.5.2.0). The above analyses were conducted using the R studio, and the graphics were prepared with Origin 2018. The effects of soil physicochemical factors on soil enzyme activity and PLFAs of soil microbes in the two soil layers of all FT treatments were analyzed by redundancy analysis (RDA)^1^. The statistical significance of the RDA was tested using a Monte Carlo permutation test (999 permutations; *P* < 0.05). Partial Mantel tests were performed to test the Spearman rank correlations between soil properties and microbial communities (The datasets of this research were attached in Supplementary Data Sheet 2).

## Results

### Effects of the Freeze–Thaw Pattern on Activity of Soil Microbial Communities

**FIGURE 1:**
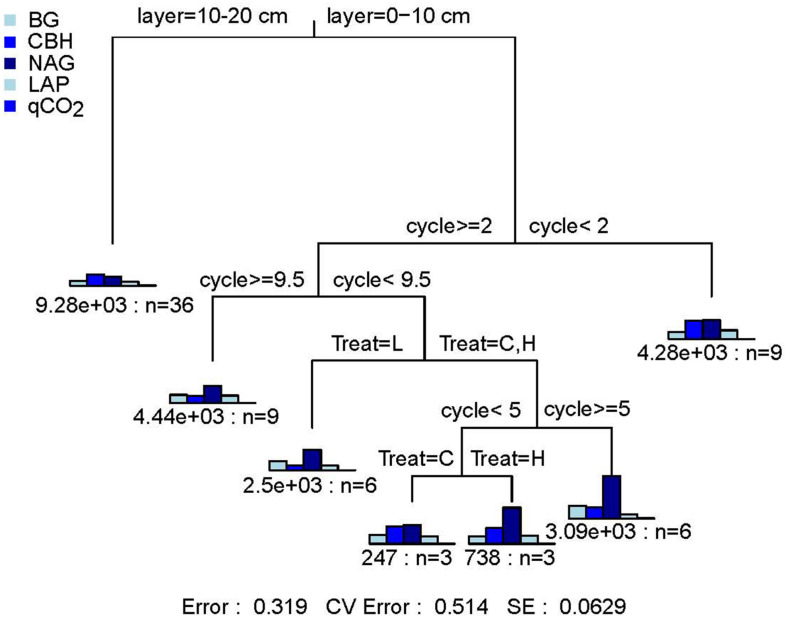
Multiple regression tree analysis of the effects of freeze–thaw times and treatment on soil microbial community activity. Error: Relative error; CV error: Cross-validation error; SE: Cross-validation standard error. Layer, soil layer; Treat, freeze-thaw temperature fluctuation; Cycle, the number of freeze-thaw cycles. BG, β-glucosidase; CBH, β-cellobiosidase; NAG, *N*-acetyl-glucosaminidase; LAP, l-leucine aminopeptidase; qCO_2_, respiration quotient. C, H, and L represent freeze-thaw treatment of 4, −4 to 4, and −14 to 4°C, respectively.

The FTF significantly affected the qCO_2_ (*P* < 0.05) and C-hydrolyzing enzyme activity (*P* < 0.05) but did not affect N-hydrolyzing enzyme activity (*P* > 0.05). The FTN significantly affected the qCO_2_ (*P* < 0.001) and C- (*P* < 0.001) and N- (*P* < 0.01) hydrolyzing enzyme activities. The three parameters were not significantly affected by the interaction of treatments (*P* > 0.05; Supplementary Table S1). According to the MRT analysis of the effects of soil layer, FTN, and FTF on microbial community activity, the three factors explained 68.1% of the variance in microbial activities (Figure 1). The microbial indicators were divided into seven groups. They were first divided by soil layer, which explained 33.6% of the change in microbial activity and then were divided by FTN and FTF, which explained 22.9 and 11.6%, respectively. The control treatment without FT (C) was a branch, and the −4 to 4°C (H) and −14 to 4°C (L) FTF treatments together composed one branch. These results showed that soil microbial activities are affected by FT and that the effects of FTN are greater than those of FTF.

### Effects of the Freeze–Thaw Pattern on Biomass and Structure of Soil Microbial Communities

**FIGURE 2:**
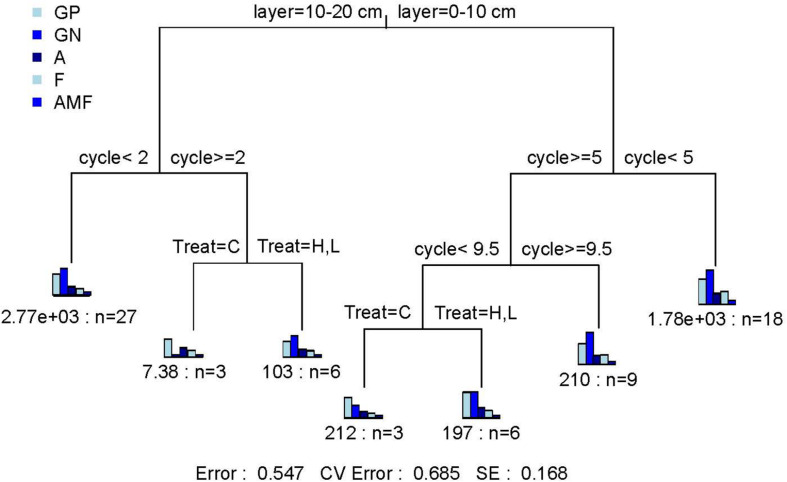
Multiple regression tree analysis of the effects of number of freeze–thaw cycles and treatment on soil microbial community structure. Error: Relative error; CV error: Cross-validation error; SE: Cross-validation standard error. Layer, soil layer; Treat, freeze-thaw temperature fluctuation; Cycle, the number of freeze-thaw cycles. GP, gram-positive bacteria; GN, gram-negative bacteria; A, actinomycetes; F, fungi; AMF, arbuscular mycorrhizal fungi. C, H, and L represent freeze-thaw treatment of 4, –4 to 4, and –14 to 4°C, respectively.

Gram-negative bacteria (GN, *P* < 0.05), fungi (F, *P* < 0.01), arbuscular mycorrhizal fungi (AMF, *P* < 0.05), and actinomycetes (Act, *P* < 0.05) were significantly affected by FTF. The FTN only significantly affected F (*P* < 0.05) and AMF (*P* < 0.05). The interaction between FTN and soil layer significantly affected tPLFAs and subgroup microorganisms, and the interaction between FTN and FTF significantly affected gram-positive bacteria (GP, *P* < 0.05), Act (*P* < 0.05), and AMF (*P* < 0.01). Notably, among subgroups of microorganisms, the AMF were sensitive to FTN (*P* < 0.05), FTF (*P* < 0.05), soil layer (*P* < 0.001), and their interactive effects [Treat^∗^Layer (*P* < 0.01); Treat^∗^Layer (*P* < 0.01); Layer^∗^Cycle (*P* < 0.001); Treat^∗^Layer^∗^Cycle (*P* < 0.001); Supplementary Table S2]. The MRT analysis showed that the three factors explained 45.3% of the variance in the biomass of microbial communities (Figure 2). The microbial community indicators were divided into six groups. They were first divided by soil layer, which explained 13.7% of the change in microbial communities, and then were divided by FTN and FTF, which explained 20.9 and 10.8%, respectively. The results indicated that soil microbial community structure is most sensitive to FTN.

### Changes in Activity of Soil Microbial Communities During the Freeze–Thaw Process

**FIGURE 3:**
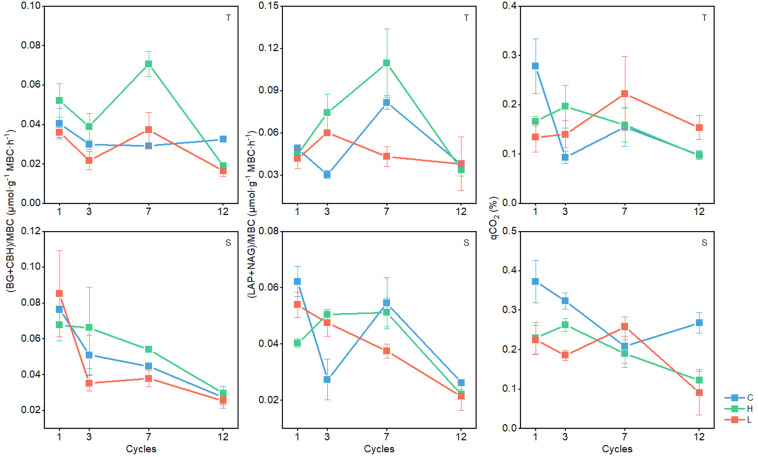
Changes in enzyme activity of soil microbial communities during freeze–thaw events in 0–10-cm (T) and 10–20-cm soil layer (S). C, H, and L represent freeze-thaw treatment of 4, –4 to 4, and –14 to 4°C, respectively.

Standardized enzyme activity is most representative of microbial physiology and is therefore valid to reflect the response of soil microbial physiology to FT events (Li et al., 2019). The qCO_2_ is an indicator of the size and activity of soil microbial populations and is considered as a measure of true microbial activities in soil (Dick et al., 2003; Jia and Xiao, 2017). In the 0–10-cm soil layer, C-hydrolyzing enzyme activities changed significantly with FTN (*P* < 0.05) in the C, H, and L treatments and generally decreased with the increase in FTN (Figure 3). In the C and H treatments, N-hydrolyzing enzyme activities and the qCO_2_ changed significantly with FTN (*P* < 0.05). With the increase in FTN, N-hydrolyzing enzyme activities first increased and then decreased, and the qCO_2_ decreased. N-hydrolyzing enzyme activities and qCO_2_ at 12th freeze-thaw cycle were significantly lower than those of the first cycle (*P* < 0.05) (Supplementary Table S3). The C-hydrolyzing enzyme activities (except for the first FT cycle), N-hydrolyzing enzyme activities (except for the third and seventh FT cycles), and the qCO_2_ (except for the seventh FT cycle) were significantly different between the FTF treatments (*P* < 0.05), but there was no consistent trend among different temperature treatments.

In the 10–20-cm soil layer, C- and N-hydrolyzing enzyme activities and the qCO_2_ were significantly influenced by FTN (*P* < 0.05) and decreased with the increase in FTN (Figure 3). C-, N-hydrolyzing enzyme activities and qCO_2_ at 12th freeze-thaw cycle were significantly lower than those of the first cycle (*P* < 0.05) (Supplementary Table S3). The C-hydrolyzing enzyme activity in the third FT cycle, N-hydrolyzing enzyme activity in the first and third FT cycles, and the qCO_2_ (except for the seventh FT cycle) were significantly different between the FTF treatments (*P* < 0.05), but there was no consistent trend among different temperature treatments.

### Changes in the Structure of Soil Microbial Communities During the Freeze–Thaw Process

**FIGURE 4:**
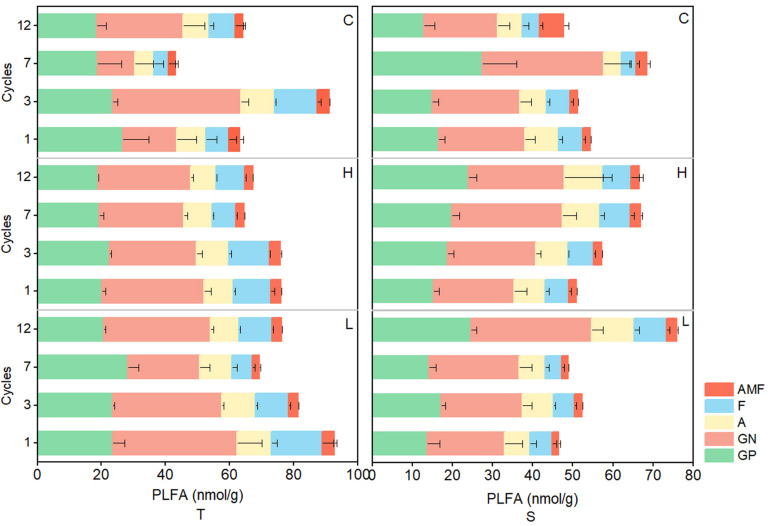
Changes in PLFAs of soil microbial communities during freeze–thaw events in 0–10-cm (T) and 10–20-cm soil layer (S). C, H, and L represent freeze-thaw treatment of 4, –4 to 4, and –14 to 4°C, respectively.

In the 0–10-cm soil layer, tPLFAs and PLFAs of microbial subgroups changed significantly and generally decreased with the increase in FTN (*P* < 0.05) in the H and L treatments, but in the 10–20-cm soil layer, the opposite trend was observed. In both soil layers, tPLFAs and PLFAs of microbial subgroups did not change consistently with the FTF treatments, and there were no significant differences (*P* > 0.05; Figure 4).

**FIGURE 5:**
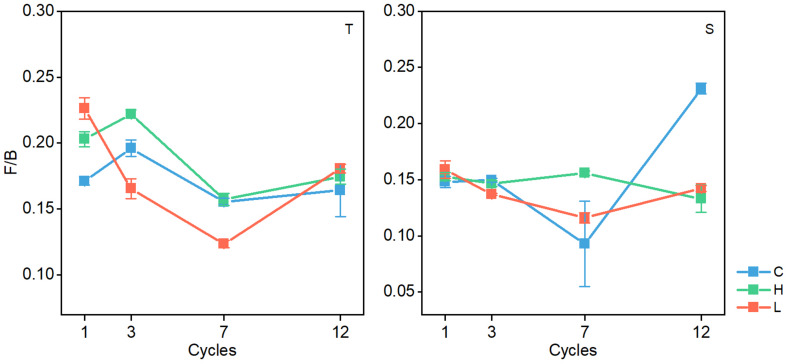
Changes in the ratio of fungal biomass to bacterial biomass in soil microbial communities during freeze–thaw events in 0–10-cm (T) and 10–20 soil layer (S). C, H, and L represent freeze-thaw treatment of 4, –4 to 4, and –14 to 4°C, respectively.

In both soil layers, the ratio of fungal PLFAs to bacterial PLFAs (F/B) changed significantly (*P* < 0.05), and the ratio generally decreased with the increase in FTN (Figure 5). The F/B ratio was significantly different among the FTF treatments (*P* < 0.05), but the difference was not consistent.

### Controls on Soil Microbial Communities and Enzyme Activities

**FIGURE 6:**
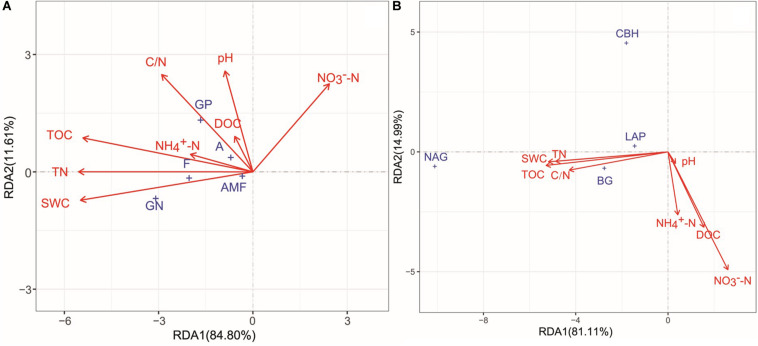
Redundancy analysis (RDA) of the relationships among soil microbial community structure **(A)**, enzyme activities **(B)**, and soil environmental factors. GP, gram-positive bacteria; GN, Gram-negative bacteria; A, actinomycetes; F, fungi; AMF, arbuscular mycorrhizal fungi. SWC, soil water content; TOC, soil total organic carbon; DOC, dissolved organic carbon; TN, total nitrogen; C/N, the ratio of total organic carbon and total nitrogen. BG, β-glucosidase; CBH, β-cellobiosidase; NAG, *N*-acetyl-glucosaminidase; LAP, l-leucine aminopeptidase.

All soil physicochemical properties combined explained 96.41 and 96.10% of the changes in microbial community structure and enzyme activities, respectively (Figure 6). According to Monte Carlo permutation tests, TN (*P* < 0.001, *R*^2^ = 0.2101), TOC (*P* < 0.001, *R*^2^ = 0.2087), SWC (*P* < 0.001, *R*^2^ = 0.2042), and C/N ratio (*P* < 0.05, *R*^2^ = 0.0962) were the major contributors to the variation observed in microbial community composition (Figure 6A). TOC (*P* < 0.001, *R*^2^ = 0.6501), TN (*P* < 0.001, *R*^2^ = 0.6129), SWC (*P* < 0.001, *R*^2^ = 0.5759), NO${}_{3}^{-}$ -N (*P* < 0.001, *R*^2^ = 0.4587), C/N (*P <* 0.001, *R*^2^ = 0.3177), DOC (*P* < 0.01, *R*^2^ = 0.1259), and NO${}_{3}^{-}$ -N (*P* < 0.05, *R*^2^ = 0.1010) significantly changed enzyme activities (Figure 6B). The results of the partial Mantel tests also showed that microbial community composition and enzyme activities were positively correlated with SWC (*P* < 0.05 and *P* < 0.01, respectively), TOC (both at *P* < 0.01), and TN (both at *P* < 0.01; Table 3).

**TABLE 3:** Spearman rank correlations (R-values of soil physicochemical variables with matrices of microbial community composition and extracellular enzyme activities based on Mantel tests).

**Soil properties**	**SWC**	**pH**	**TOC**	**DOC**	**TN**	**NH${}_{4}^{+}$ -N**	**NO${}_{3}^{-}$ -N**	**C/N**
Microbial community composition	0.11*	−0.01	0.17**	−0.03	0.17**	−0.12	−0.00	−0.02
Enzyme activities	0.29**	−0.03	0.40**	0.06	0.32**	−0.11	−0.17**	0.20

*P < 0.05; **P < 0.01.

## Discussion

### Effects of Freeze–Thaw Patterns on Soil Microbial Community Structure and Activities

According to the repeated measures ANOVA, the FTN significantly affected microbial activities (*P* < 0.001). In addition, tPLFAs and the biomass of various microbial groups were significantly affected by the interaction between FTN and soil layer (*P* < 0.05). According to the MRT analysis, the FTN explained more of the variation in microbial activities and microbial community structure than the FTF (Figures 1, 2). The results may be explained by soil weathering that occurs in the FT process. Geologically, the interactions between liquid water, solid ice, and water vapor exchange mass and energy with the external environment is called a weathering process. Freezing is a weathering process, but the effects are potentially more severe than those of common physical weathering and the intensity of frost weathering is proportional to the FTN (Zhang et al., 2016). An increase in FTN results in more severe damage to soil structure by weathering and increases the redistribution of mineral elements, resulting in a greater effect on the soil microbial community (Jefferies et al., 2010).

In order to demonstrate the universality of the above conclusions, the literatures on the tests of FTN and FTF were examined to determine the effects of the two treatment factors on microbial biomass, soil mineralization rate, and the substrate related to soil microbial metabolism (such as TC, TN, and DOC). Repeated measures ANOVA and MRT analysis were conducted on the data obtained (details are shown in Supplementary Tables S4–S10 and Supplementary Figures S3–S8). On the basis of the analyses, the results of most studies supported the conclusions of the present study, i.e., compared with FTF, FTN has a greater effect on the relevant characteristics and processes of soil microorganisms. With the increase in FTN, the cells are lysed, and the carbon and nutrients inside the cells dissolve, which increases the amounts of soil nutrients that can be leached (Joseph and Henry, 2008; Sonja et al., 2015; Du et al., 2020). The release of these nutrients provides energy and nutrient sources for the surviving microorganisms to metabolize and grow (Koponen et al., 2006). In this condition, in soils with large amounts of water transfer, frequent FT events lead to large amounts of nutrient loss (Henry, 2008). Therefore, the increase in FTN caused by the absence of snow in future winters will change soil nutrient contents. Although it is believed that laboratory incubations may not be accurate enough to assess the effects on soil nutrient cycling, a meta-analysis by Song et al. (2017) shows that in both laboratory incubations and *in situ* observation experiments, FT events increase the dissolution of soil carbon and nutrients.

In conclusion, as hypothesized, increases in FTN and FTF caused by climate warming in the Daxing’an Mountains would have different effects on soil microbial activity and community structure, with the frequency of FT events having the greatest effect on properties of soil microbial communities. Notably, the effects of FT patterns on microbial communities generally depend on the forest type and the soil environment. Therefore, the responses of soil microbial communities to FT patterns need to be further investigated at different latitudes and in different ecosystems and soil types.

### Changes in Soil Microbial Activity and Community Structure During Freeze–Thaw Events

In this study, C- and N-hydrolyzing enzyme activities and the qCO_2_ were negatively affected by the number of FT cycles (Figure 3), which indicated that an increase in FTN could inhibit microbial activities, a result consistent with that of most studies (Han et al., 2017; Kim et al., 2018; Ren et al., 2018). Water swelling in the soil caused by repeated FT events disrupts the osmotic pressure balance between inside and outside of cells, which inhibits physiological functions (Buford Price and Todd, 2004) and results in a decline in microbial activity.

The FTN significantly affected the biomass of microbial subgroups (*P* < 0.05), and the soil layer was an important variable in determining the effects of FT patterns on soil microbial biomass. In the 0–10-cm soil layer, soil microbial biomass decreased with the increase in FTN, whereas in the 10–20-cm soil layer, the opposite was observed (Figure 4). Freezing destroys the physiological functions of the cytoplasm and leads to cell death, reducing microbial biomass (Dawson, 1994). However, owing to the heat-insulating properties of surface soil (resulting in a thermal gradient with soil depth), the temperatures in deeper soil generally fluctuate less, resulting in less damage to microbial cells (Han et al., 2018). Thus, the response pattern of soil microorganisms to FTN is spatially heterogeneous. In addition, the interaction between soil layer and FTN also significantly affected soil tPLFAs and subgroups of microorganisms (*P* < 0.05). These results may due to the redistribution of water and salt and the changes in pH, redox potential, and oxygen in different soil layers after frequent FT cycles (Hu et al., 2015), which cause microbial niche differentiation.

All subgroups microorganisms showed similar trends with the increase in FTN but differed in sensitivity to FT events as previously observed (Kreyling et al., 2012). Among the microbial groups, the AMF were the most sensitive to FTN, FTF, soil layer, and their interactions. In addition to the direct effects of FT, FT events lead to the destruction of root cells because of frost heaving (Tierney et al., 2001; Grogan et al., 2004) and to a substantial reduction in the biomass of living fine roots (Reinmann and Templer, 2015), negatively affecting the symbiosis of AMF with plant roots. AMF are ubiquitous symbionts in terrestrial ecosystems, and glomalin-related soil protein released from hyphae and spores is one of the most stable carbon components in soil and can promote the chelation of organic carbon (Wang et al., 2018). Therefore, the decrease in AMF with the increase in FTN may reduce the stability of soil carbon. According to Xiao et al. (2019), FT events reduce the soil glomalin-related soil protein content and thereby reduce sequestration of soil organic carbon in a forest ecosystem.

The F/B ratio can also characterize the level of soil C sequestration and the stability of the soil carbon pool (Yergeau et al., 2012; Malik et al., 2016; Liu et al., 2019). An increase in the F/B ratio indicates an improvement in soil C sequestration capacity and ecosystem stability (Boyle et al., 2008; Ou et al., 2019). In the present study, the F/B ratio decreased significantly when the FTN increased (Figure 5), indicating that FT events can reduce the sequestration of soil carbon and the stability of the carbon pool. In previous studies, researchers found that frequent FT events reduce soil carbon accumulation by directly measuring the soil carbon concentration during FT events (Henry, 2007; Wang et al., 2015; Tang et al., 2016; Xiao et al., 2019). By analyzing the response of AMF and F / B to FT, this study provides an explanation based on the analysis of microbial communities.

### Factors That Drive Changes in Soil Microbial Communities and Enzyme Activities During Freeze–Thaw Events

The above results show that the FT patterns affected the properties of soil microbial communities. Freeze–thaw events change the soil physicochemical properties by destroying the soil structure and affecting the soil water and heat conditions (Ren et al., 2018). Such changes further affect the formation of microbial habitats and niches (Feng et al., 2007; Haei et al., 2011) and are linked to the transformations of carbon and nutrients (Jansson and Hofmockel, 2020). Consistent with the results of most studies, in this study, soil SWC, TOC, and TN were the major factors affecting microbial communities and were positively correlated with soil microbial biomass and community activity (Figure 6 and Table 3), indicating that water and substrate supply were the major factors affecting microorganisms during the FT process (Bölter et al., 2005; Sorensen et al., 2018). FT events alter the quality, quantity, and turnover of soil organic matter and the availability of water to microbial communities (Haei et al., 2011), resulting in the decreases water use efficiency and substrate supply that limit microbial activity (Sorensen et al., 2018). By contrast, in soils rich in organic matter, high nutrient availability and stable physical conditions are benefit for microbial metabolism (Bragazza et al., 2019), which result in a higher rates of recovery microorganisms after stress (LagerlöF et al., 2014) and lower loss of microbial biomass (Yanai et al., 2004; Zhang et al., 2017). Yanai et al. (2004) examined soil microbial biomass and found a significantly positive correlation between the rate of microbial survival and organic matter content of soil among different land use types in temperate regions after four consecutive FT cycles. Han et al. (2017) also found that soil organic matter was a key factor in the rapid recovery of soil microorganisms following repeated FT events in semi-arid areas of the Loess Plateau. Therefore, because of the differences in soil biological and abiotic characteristics in different ecosystems, the resistance of soil microorganisms to FT events likely depends largely on the soil water and nutrient conditions of the ecosystem. This dependence suggests that sufficient water and nutrients can limit the damage to microorganisms caused by FT events (Yanai et al., 2004). Thus, the status of soil water and fertilizer consequently provides a reference for predicting the response of soil microorganisms to FT events in different ecosystems. Of course, the next research goal is to study the responses of soil microorganisms to freezing and thawing when conditions are artificially controlled.

FT events destroy microbial cells, causing death, and the phospholipids in cells are rapidly decomposed. The PLFA profile provides the biomass of living microbes in the soil based on the content of each component of PLFAs, and therefore is widely used to study the responses of soil microorganisms to FT events. However, the low resolution of this method makes understanding the microbial responses at the species level difficult. Molecular biotechnology (e.g., next generation sequencing) can provide more detailed taxonomic information, but because of the longer degradation period of nucleic acids in dead microorganisms than that of PLFAs, molecular biotechnology is relatively poor in reflecting the environmental sensitivity of microorganisms (Frostegård et al., 2011). Therefore, because of the short period of FT events in this study, PLFAs were used to analyze the microbial community. Our research provided a framework to study the responses of soil microorganisms to FT patterns. In the future, suitable or combine multiple methods should be chosen according to the research purpose to obtain more complete and detailed information on microbial communities, with the goal to more exactly interpret the contributions of subgroups of microorganisms to enzyme activity.

## Conclusion

The effects of FTN and FTF on soil microbial communities were different in the soil of a *L. gmelinii* forest. The frequency of FT events had the greatest effect on soil microbial activity, microbial biomass, and microbial community structure. Frequent FT cycles decreased the biomass of microbial subgroups and the activity of microbial communities. In addition, the response of the biomass of microbial subgroups to FT events was vertically heterogeneous. Among all microbial groups, the AMF were the most sensitive to FTN, FTF, soil layer, and their interactions. When the negative effects on AMF are combined with the decrease in F/B ratio with the increase in FTN, a microbial-based explanation is provided for the reduction in soil carbon sequestration caused by frequent FT events. Soil SWC, TOC, and TN were the major factors affecting microbial communities. Therefore, the resistance of soil microorganisms to FT events depends largely on the soil water and nutrient conditions of the ecosystem, and sufficient water and nutrient can limit the damage to microorganisms caused by FT events. Our results will lead to better predictions of the likely effects of future climate change on soil microorganisms.

## Data Availability Statement

All datasets generated for this study are included in the Data Sheet of Supplementary Material.

## Author Contributions

FF, TC, and ML designed the project. ML and ST carried out the experiments. FF and ML participated in the analysis and wrote the manuscript. All authors read and approved the final manuscript.

## Conflict of Interest

The authors declare that the research was conducted in the absence of any commercial or financial relationships that could be construed as a potential conflict of interest.

## Footnotes

1 www.omicshare.com/tools/
