# Supplementary material for: Soil Microbial Community Response Differently to the Frequency and Strength of Freeze–Thaw Events in a Larix gmelinii Forest in the Daxing’an Mountains, China
Source: Front Microbiol. 2020 Jun 3;11:1164. doi: 10.3389/fmicb.2020.01164 (PMC7283528; doi:10.3389/fmicb.2020.01164)
Supplement: Supplementary file 1 [file Data_Sheet_1.docx]

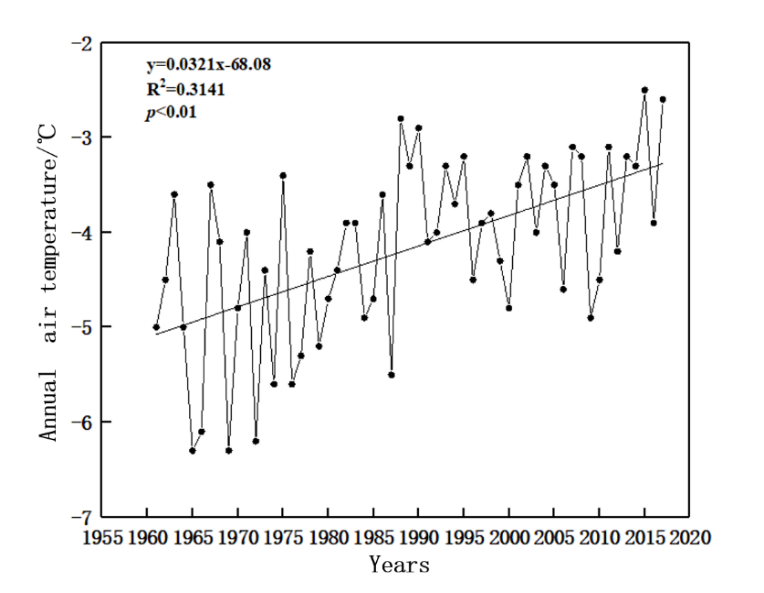
Supplementary Material


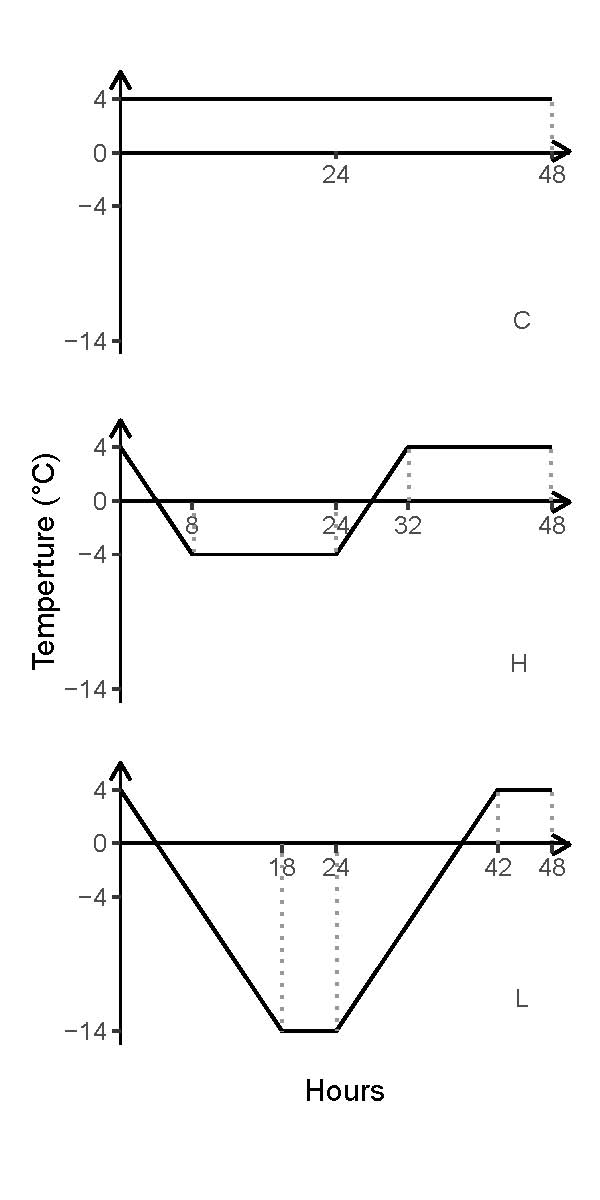
**Figure S1** Changes of annual average temperature in Mohe from 1961 to 2017

**Figure S2** A freeze-thaw cycle of three freeze-thaw temperature treatments of constant 4°C (C), FT cycles between −4°C and 4°C (H) and (3) FT cycles between −14°C and 4°C (L)

**Table S1** Multivariate variance analysis of the effect of freeze-thaw pattern to soil microbial community activity

|  | Df | qCO_2_ | |  | (BG+CBH)/MBC | |  | (LAP+NAG)/MBC | |
| --- | --- | --- | --- | --- | --- | --- | --- | --- | --- |
|  |  | *F* | *P* |  | *F* | *P* |  | *F* | *P* |
| FTF | 2 | 4.039 | 0.023* |  | 3.181 | 0.049 * |  | 1.169 | 0.318 |
| FTN | 1 | 15.364 | <0.001*** |  | 39.628 | < 0.001*** |  | 8.179 | 0.006** |
| Layer | 1 | 12.699 | <0.001*** |  | 12.643 | < 0.001*** |  | 8.971 | 0.004** |
| FTF*layer | 2 | 3.330 | 0.043* |  | 0.383 | 0.683 |  | 1.307 | 0.278 |
| FTF*cycle | 2 | 1.545 | 0.222 |  | 0.482 | 0.620 |  | 0.875 | 0.422 |
| layer*cycle | 1 | 1.520 | 0.222 |  | 6.200 | 0.016* |  | 1.269 | 0.265 |
| FTF*layer*cycle | 2 | 0.873 | 0.423 |  | 0.502 | 0.608 |  | 0.202 | 0.817 |

**Table S2** Multivariate variance analysis of the effect of freeze-thaw pattern on soil microbial community

|  |  | GP | |  | GN | |  | Act | |  | F | |  | AMF | |  | tPLFA | |
| --- | --- | --- | --- | --- | --- | --- | --- | --- | --- | --- | --- | --- | --- | --- | --- | --- | --- | --- |
|  | Df | *F* | *p* |  | *F* | *p* |  | *F* | *p* |  | *F* | *p* |  | *F* | *p* |  | *F* | *p* |
| FTF | 2 | 0.321 | 0.727 |  | 3.221 | 0.047 * |  | 6.741 | 0.002 ** |  | 4.014 | 0.023 * |  | 4.171 | 0.020 * |  | 2.404 | 0.099 |
| FTN | 1 | 0.275 | 0.602 |  | 1.388 | 0.244 |  | 0.477 | 0.493 |  | 5.088 | 0.028 * |  | 4.028 | 0.049 * |  | 0.105 | 0.747 |
| Layer | 1 | 13.904 | <0.001*** |  | 9.574 | 0.003** |  | 16.667 | <0.001*** |  | 45.470 | <0.001*** |  | 13.702 | <0.001*** |  | 21.668 | <0.001*** |
| FTF*Layer | 2 | 2.610 | 0.082 |  | 0.410 | 0.666 |  | 2.371 | 0.102 |  | 0.794 | 0.457 |  | 5.884 | 0.005 ** |  | 1.456 | 0.241 |
| FTF*FTN | 2 | 3.582 | 0.034 * |  | 0.316 | 0.731 |  | 3.205 | 0.048 * |  | 0.148 | 0.863 |  | 6.637 | 0.002 ** |  | 0.128 | 0.880 |
| layer*FTN | 1 | 12.259 | <0.001*** |  | 5.559 | 0.022 * |  | 8.553 | 0.005 ** |  | 7.935 | 0.007 ** |  | 41.710 | <0.001*** |  | 12.825 | <0.001*** |
| FTF*Layer*FTN | 2 | 0.181 | 0.835 |  | 0.397 | 0.673 |  | 2.560 | 0.086 |  | 1.949 | 0.151 |  | 10.628 | <0.001*** |  | 0.377 | 0.687 |

Significance levels: *, *p*<0.05; **, *p*<0.01; ***, *p*<0.001.

**Table S3** Analysis of least-significant difference the effect of freeze-thaw pattern on subgroup of microbial community activity

| Soil layer | Cycles | qCO_2_ | | |  | (BG+CBH)/MBC | | |  | (LAP+NAG)/MBC | | | |
| --- | --- | --- | --- | --- | --- | --- | --- | --- | --- | --- | --- | --- | --- |
|  |  | C | H | L |  | C | H | L |  | C | H | L |  |
| T | 1 | Aa | ABb | Ab |  | Aa | Aa | Aa |  | Aa | ACa | Aa |  |
|  | 3 | Ba | Ab | Aab |  | Bab | Ba | Bb |  | Ba | Ab | Ab |  |
|  | 7 | Ba | ABa | Aa |  | Ba | Cb | Aa |  | Ca | Ba | Ab |  |
|  | 12 | Ba | Ba | Ab |  | Ba | Db | Bb |  | Da | Ca | Aa |  |
| S | 1 | Aa | Ab | Ab |  | Aa | Aa | Aa |  | Aa | Ab | Aa |  |
|  | 3 | ABa | Ab | Ac |  | Ba | Aa | Ba |  | Aa | Bb | Ab |  |
|  | 7 | Ca | ABa | Aa |  | Ba | ABb | Bc |  | Ba | Bab | Bb |  |
|  | 12 | BCa | Bb | Bb |  | Ca | Ba | Ba |  | Ca | Ca | Ca |  |

The lower case letters represent the significant difference between freeze-thaw temperature fluctuations, and the upper case letters represent the significant difference in freeze-thaw cycle times

**Table S4** Studies of the effect of soil freeze-thaw on soil properties

| No. | Reference | Interpretation amounts (%) | | Results shown in | System | Topic | Experiment design | |
| --- | --- | --- | --- | --- | --- | --- | --- | --- |
|  |  | FTN | FTF |  |  |  | Freeze-thaw FTF | Number of cycles |
| Paper 1 | (Wang *et al.*, 2015) | 40.93 | - | Table S5, Figure S3 | Peatlands | WEOC, MBC, Amylase, Celluase, Invertase | -5 – 5℃  -10 – 10℃ | 0, 3, 5, 10, 15 |
| Paper 2 | (Wang *et al.*) | 29.95 | 6.43 | Table S6, Figure S4 | Light cyanobacterial soil crusts, dark cyanobacterial soil crusts | TC, TN, Hydraulic conductivity, strength | -5 – 10℃  -10 – 10℃ | 0, 1, 2, 3, 4, 5, 6 |
| Paper 3 | (Zhou *et al.*) | 72.39 (a) 46.46(b) 72.52(c) 64.90 (d) | 22.70 (a)  33.02 (b)  19.09 (c)  24.29 (d) | Table S7, Figure S5 | broad-leaved Korean pine forest (Zheng *et al.*), spruce-fir forest (SF), *Betula ermanii* forest (EB), subalpine tundra (ST) | NH_4_^+^-N, NO_3_^-^-N, N mineralization rate | -5 – 5℃  -25 – 5 | 0, 1, 3, 7, 15 |
| Paper 4 | (Hentschel *et al.*, 2008) | 4.05(a)  33.19(b) | 16.76(a)  - | Table S8, Figure S6 | Norway spruce (*Picea abies*) forest | NH_4_^+^-N, NO_3_^-^-N, TN, DOC, Specific UV absorbance 280 nm, Humification index | constant 5℃  -3 – 5℃  -8 – 5℃  -13 – 5℃ | 1, 2, 3 |
| Paper 5 | (Sulkava & Huhta, 2003) | 82.24 | - | Table S9, Figure S7 | silviculturally managed Scots pine (*Pinus sylvestris*) stand of the Vaccinium type | Nematodes, MB, Basal respiration, NH_4_^+^-N, CO_2_ evolution | constant -2℃; -2 – 2℃ for 1 cycle; -2 – 2℃ for 2 cycles then -16 – 2 ℃ for 2 cycles | |
| Paper 6 | (Kreyling *et al.*, 2012) | 13.36(a)  43.69(b) | -  48.63(b) | Table S10, Figure S8 |  | Root damage, relative electrolyte leakage (REL) | -5 – 5℃ for 1 cycle; -10 – 5℃ for 1 cycle; -5 – 5℃ for 3 cycles | |

**Data collection:** The Web of Science was used to search for peer-reviewed journal articles published between 2000 and 2019. The search verified that FTN and FTF were both used as experimentally controlled variables to study the effect of simulated FT on soil properties. The data were compiled directly from tables and figures using Get Data Graph Digitizer software (version 2.26). The same analyses used in the present study were used to analyze the data to ensure the comparability of the results.

**Table S5** Multivariate variance analysis of the effect of freeze-thaw pattern on soil properties and enzyme in Paper 1

|  |  | WEOC | |  | MBC | |  | Amylate | |  | Celluase | |  | Invertase | |
| --- | --- | --- | --- | --- | --- | --- | --- | --- | --- | --- | --- | --- | --- | --- | --- |
|  | Df | *F* | *p* |  | *F* | *p* |  | *F* | *p* |  | *F* | *p* |  | *F* | *p* |
| FTF | 1 | 8.467 | 0.005** |  | 3.091 | 0.083 |  | 80.535 | <0.001*** |  | 0.015 | 0.904 |  | 6.908 | 0.010 * |
| FTN | 1 | 17.757 | <0.001*** |  | 26.109 | <0.001*** |  | 63.316 | <0.001*** |  | 16.469 | <0.001*** |  | 21.572 | <0.001*** |
| Layer | 2 | 55.972 | <0.001*** |  | 40.539 | <0.001*** |  | 1213.387 | <0.001*** |  | 388.986 | <0.001*** |  | 659.945 | <0.001*** |
| FTF*FTN | 1 | 0.006 | 0.938 |  | 0.018 | 0.895 |  | 0.619 | 0.434 |  | 3.182 | 0.078 |  | 0.003 | 0.957 |
| FTF*Layer | 2 | 0.840 | 0.436 |  | 0.108 | 0.897 |  | 6.274 | 0.003** |  | 13.457 | <0.001*** |  | 2.274 | 0.110 |
| layer*FTN | 2 | 1.631 | 0.202 |  | 0.285 | 0.753 |  | 3.827 | 0.026 |  | 2.797 | 0.067 |  | 1.638 | 0.201 |
| FTF*Layer*FTN | 2 | 0.029 | 0.972 |  | 0.548 | 0.580 |  | 9.521 | <0.001*** |  | 5.775 | 0.005** |  | 0.952 | 0.391 |

WEOC, water-extracted organic carbon, MBC, microbial biomass carbon.


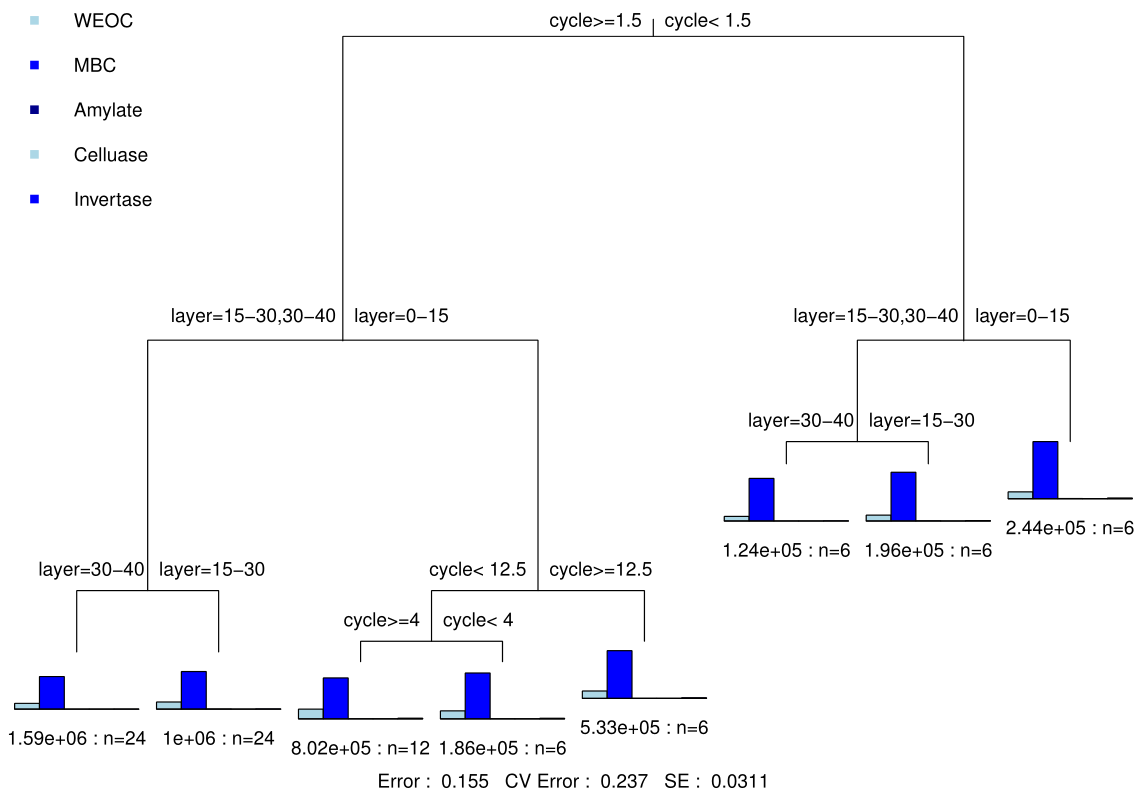


**Figure S3** Multiple Regression Tree analyzes of the impact of freeze-thaw pattern to soil properties and enzyme in Paper 1. Layer: 43.55%; FTN: 40.93%.

**Table S6** Multivariate variance analysis of the effect of freeze-thaw pattern on soil properties in Paper 2

|  |  | TC | |  | TN | |  | TC/TN | |  | Hydraulic conductivity | |  | Strength | |
| --- | --- | --- | --- | --- | --- | --- | --- | --- | --- | --- | --- | --- | --- | --- | --- |
|  | Df | *F* | *p* |  | *F* | *p* |  | *F* | *p* |  | *F* | *p* |  | *F* | *p* |
| FTF | 1 | 33.815 | <0.001*** |  | 22.704 | <0.001*** |  | 3.957 | 0.022* |  | 12.277 | <0.001*** |  | 16.918 | <0.001*** |
| FTN | 1 | 439.689 | <0.001*** |  | 573.621 | <0.001*** |  | 219.866 | <0.001*** |  | 8.973 | 0.003** |  | 5.007 | 0.027 * |
| Type | 2 | 2522.674 | <0.001*** |  | 5718.133 | <0.001*** |  | 643.787 | <0.001*** |  | 1841.167 | <0.001*** |  | 355.935 | <0.001*** |
| FTF*FTN | 1 | 14.064 | <0.001*** |  | 1.607 | 0.205 |  | 1.970 | 0.144 |  | 8.223 | <0.001*** |  | 18.508 | <0.001*** |
| FTF*Type | 2 | 12.163 | <0.001*** |  | 5.916 | 0.004** |  | 4.840 | 0.010** |  | 13.702 | <0.001*** |  | 6.492 | 0.002 |
| Type*FTN | 2 | 95.720 | <0.001*** |  | 4.136 | 0.044* |  | 216.770 | <0.001*** |  | 10.267 | 0.002** |  | 8.095 | 0.005 |
| FTF*Type*FTN | 2 | 0.948 | 0.39 |  | 2.395 | 0.096 |  | 1.579 | 0.211 |  | 14.978 | <0.001*** |  | 13.890 | <0.001*** |

Type: Soil type.


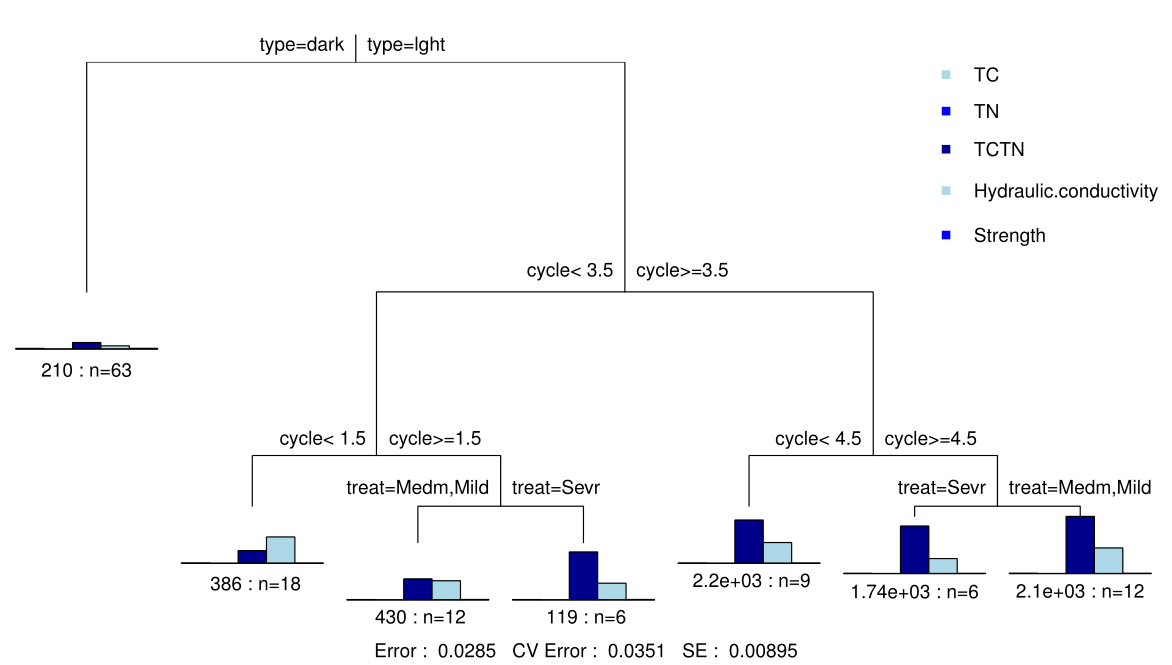


**Figure S4** Multiple Regression Tree analyzes of the impact of freeze-thaw pattern to soil properties in Paper 2. FTN: 29.95%; FTF: 6.43%; soil type: 60.77%.

**Table S7** Multivariate variance analysis of the effect of freeze-thaw pattern on soil properties in different forest types in Paper 3

|  |  |  | SF | |  | BK | |  | BE | |  | ST | |
| --- | --- | --- | --- | --- | --- | --- | --- | --- | --- | --- | --- | --- | --- |
|  |  | Df | *F* | *p* |  | *F* | *p* |  | *F* | *p* |  | *F* | *p* |
| NH_4_^+^-N | FTF | 2 | 199.994 | <0.001*** |  | 607.08 | <0.001*** |  | 170.340 | <0.001*** |  | 130.385 | <0.001*** |
|  | FTN | 1 | 1208.577 | <0.001*** |  | 629.10 | <0.001*** |  | 789.736 | <0.001*** |  | 501.368 | <0.001*** |
|  | W | 1 | 38.833 | <0.001*** |  | 102.70 | <0.001*** |  | 13.692 | <0.001*** |  | 28.239 | <0.001*** |
|  | FTF*FTN | 2 | 168.328 | <0.001*** |  | 454.88 | <0.001*** |  | 105.312 | <0.001*** |  | 83.339 | <0.001*** |
|  | FTF*W | 2 | 0.007 | 0.931 |  | 34.52 | <0.001*** |  | 1.060 | 0.308 |  | 2.466 | 0.122 |
|  | W*FTN | 1 | 38.809 | <0.001*** |  | 81.76 | <0.001*** |  | 10.555 | 0.002** |  | 32.218 | <0.001*** |
|  | FTF*W*FTN | 2 | 0.778 | 0.382 |  | 43.93 | <0.001*** |  | 5.606 | 0.022* |  | 10.749 | 0.002** |
| NO_3_^-^-N | FTF | 2 | 5.579 | 0.022* |  | 2.100 | 0.153 |  | 7.512 | 0.008 ** |  | 0.050 | 0.823 |
|  | FTN | 1 | 2.254 | 0.139 |  | 207.568 | <0.001*** |  | 0.264 | 0.609 |  | 7.299 | 0.009*** |
|  | W | 1 | 17.351 | <0.001*** |  | 76.142 | <0.001*** |  | 19.645 | <0.001*** |  | 30.754 | <0.001*** |
|  | FTF*FTN | 2 | 0.266 | 0.608 |  | 21.439 | <0.001*** |  | 0.003 | 0.957 |  | 2.248 | 0.140 |
|  | FTF*W | 2 | 9.988 | 0.003** |  | 4.130 | 0.047* |  | 5.715 | 0.020* |  | 1.853 | 0.179 |
|  | W*FTN | 1 | 22.717 | <0.001*** |  | 136.151 | <0.001*** |  | 0.136 | 0.714 |  | 4.651 | 0.036* |
|  | FTF*W*FTN | 2 | 6.778 | 0.012* |  | 0.546 | 0.463 |  | 0.000 | 0.983 |  | 2.144 | 0.149 |
| N mineralization rate | FTF | 2 | 82.689 | <0.001*** |  | 17.843 | 0.003** |  | 5.726 | 0.044* |  | 30.350 | <0.001*** |
|  | FTN | 1 | 37.258 | <0.001*** |  | 7.091 | 0.029 |  | 12.961 | 0.007** |  | 32.750 | <0.001*** |
|  | W | 1 | 1.329 | 0.283 |  | 2.248 | 0.172 |  | 4.322 | 0.071 |  | 1.521 | 0.252 |
|  | FTF*FTN | 2 | 6.063 | 0.039* |  | 2.835 | 0.131 |  | 0.378 | 0.556 |  | 3.435 | 0.101 |
|  | FTF*W | 2 | 0.030 | 0.866 |  | 1.629 | 0.238 |  | 0.479 | 0.509 |  | 0.089 | 0.773 |
|  | W*FTN | 1 | 0.641 | 0.446 |  | 1.491 | 0.257 |  | 1.923 | 0.203 |  | 0.114 | 0.744 |
|  | FTF*W*FTN | 2 | 0.435 | 0.528 |  | 0.268 | 0.619 |  | 0.677 | 0.434 |  | 1.404 | 0.270 |

W: soil water content.


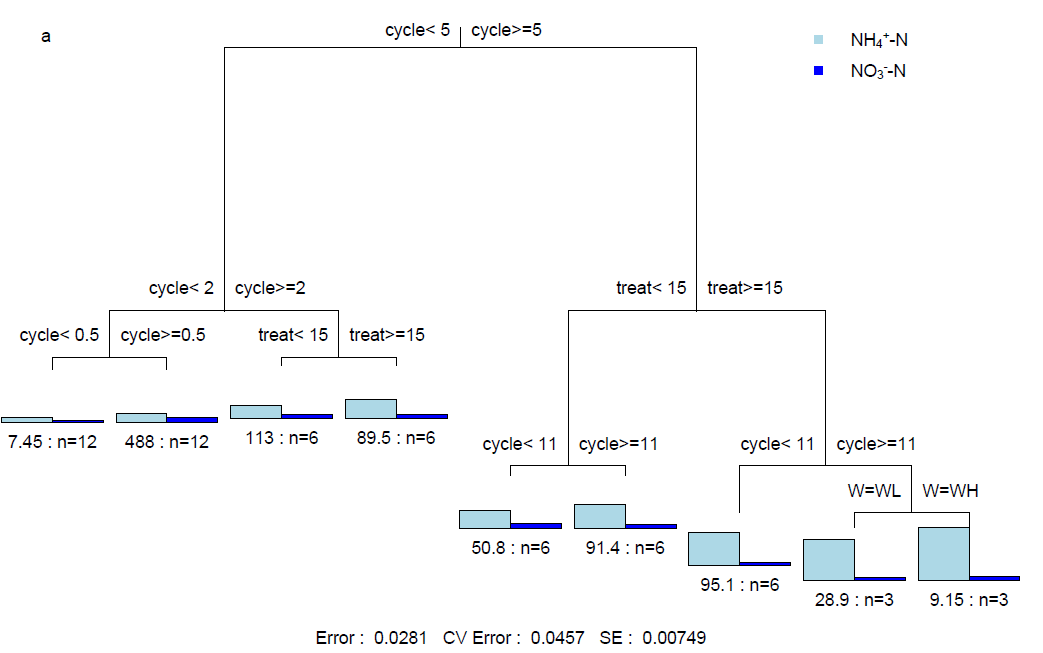

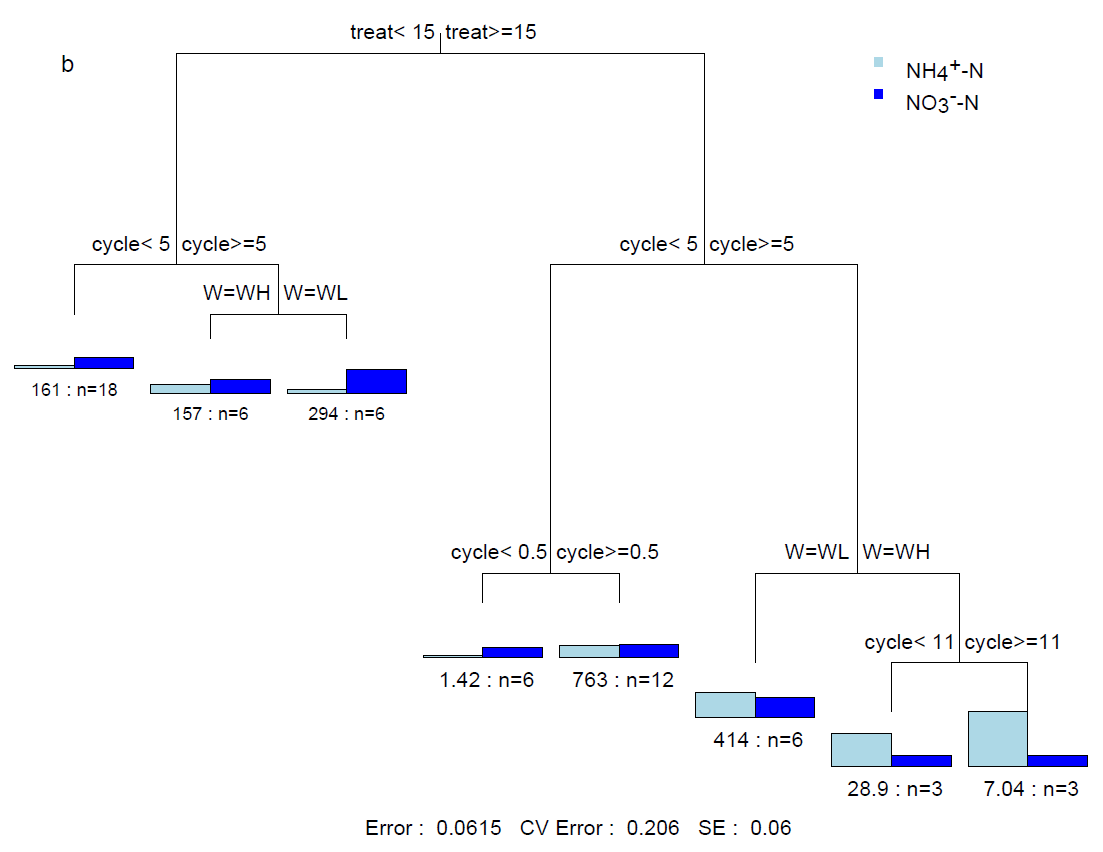


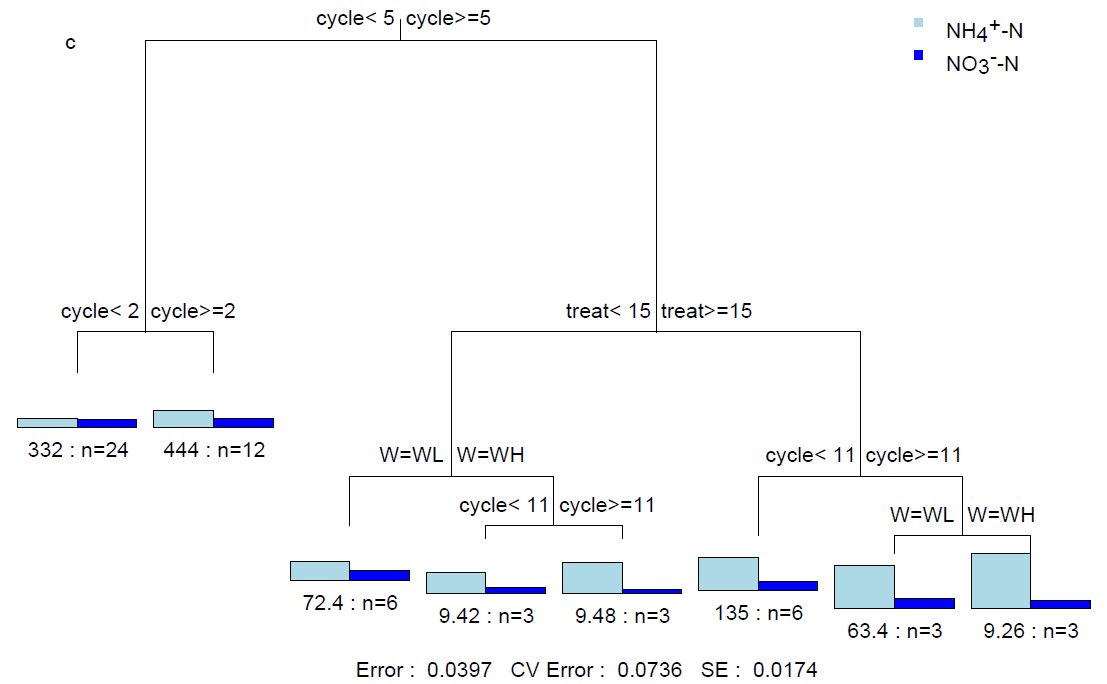

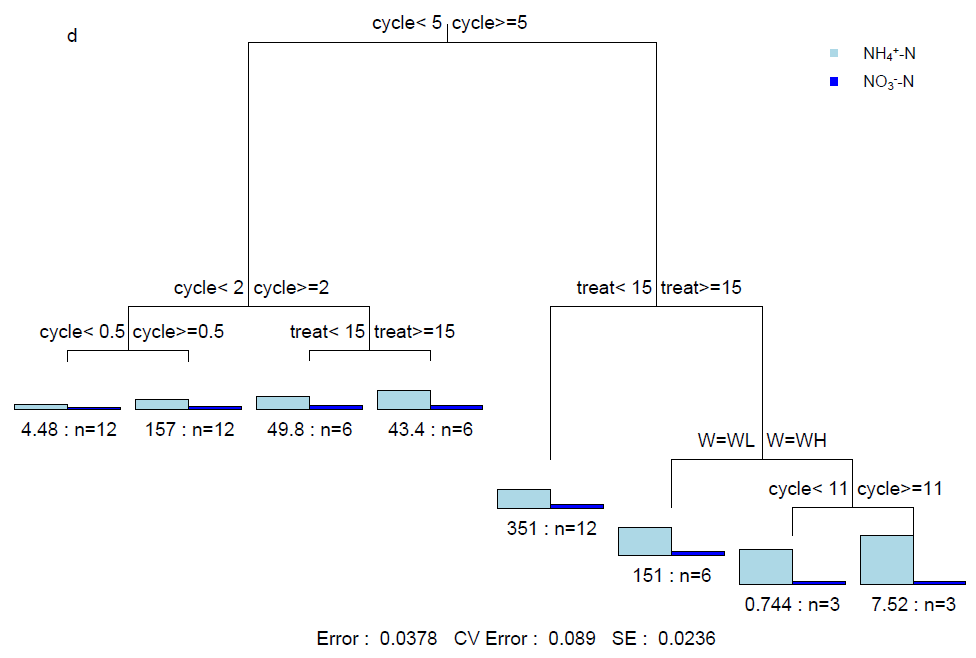


**Figure S5** Multiple Regression Tree analyzes of the impact of freeze-thaw pattern to soil NH_4_^+^-N and NO_3_^-^-N in Paper 3. a: BE, FTN: 72.39%; FTF: 22.70%; W:2.10 %; b: BK, FTN: 46.46%; FTF: 33.02%; W: 14.38%; c: SF, FTN: 72.52%; FTF: 19.09%; W: 4.41%; d: ST, FTN: 64.90%; FTF: 24.29%; W: 7.03%.

**Table S8** Multivariate variance analysis of the effect of freeze-thaw pattern on soil properties in Paper 4

|  |  | Leaching loss of NH_4_^+^-N | |  | Leaching loss of NO_3_^-^-N | |  | Leaching loss of TN | |  | Leaching loss of DOC | |
| --- | --- | --- | --- | --- | --- | --- | --- | --- | --- | --- | --- | --- |
|  | Df | *F* | *p* |  | *F* | *p* |  | *F* | *p* |  | *F* | *p* |
| FTF | 1 | 18.670 | <0.001*** |  | 87.422 | <0.001*** |  | 49.809 | <0.001*** |  | 9.701 | 0.003** |
| FTN | 1 | 38.785 | <0.001*** |  | 62.753 | <0.001*** |  | 63.831 | <0.001*** |  | 0.658 | 0.420 |
| SM | 1 | 441.650 | <0.001*** |  | 0.012 | 0.914 |  | 68.538 | <0.001*** |  | 269.685 | <0.001*** |
| FTF*FTN | 1 | 6.175 | 0.016 |  | 3.870 | 0.054 |  | 5.505 | 0.022* |  | 1.039 | 0.312 |
| FTF*SM | 1 | 33.112 | <0.001*** |  | 22.296 | <0.001*** |  | 31.784 | <0.001*** |  | 3.152 | 0.081 |
| SM*FTN | 1 | 16.613 | <0.001*** |  | 14.244 | <0.001*** |  | 2.588 | 0.113 |  | 1.312 | 0.256 |
| FTF*SM*FTN | 1 | 10.373 | 0.002** |  | 0.032 | 0.858 |  | 1.302 | 0.258 |  | 0.239 | 0.636 |

|  |  | Specific UV absorbance 280 nm | |  | Humification index | |
| --- | --- | --- | --- | --- | --- | --- |
|  | Df | *F* | *p* |  | *F* | *p* |
| FTF | 3 | 1.751 | 0.169 |  | 4.694 | 0.005 |
| FTN | 1 | 33.832 | <0.001*** |  | 96.253 | <0.001*** |
| Time | 1 | 49.995 | <0.001*** |  | 210.842 | <0.001*** |
| FTF*FTN | 3 | 11.212 | <0.001*** |  | 3.621 | 0.018 |
| FTF*Time | 3 | 1.730 | 0.173 |  | 2.285 | 0.089 |
| Time*FTN | 1 | 0.008 | 0.929 |  | 63.315 | <0.001*** |
| FTF*Time*FTN | 3 | 1.757 | 0.167 |  | 3.834 | 0.014 |


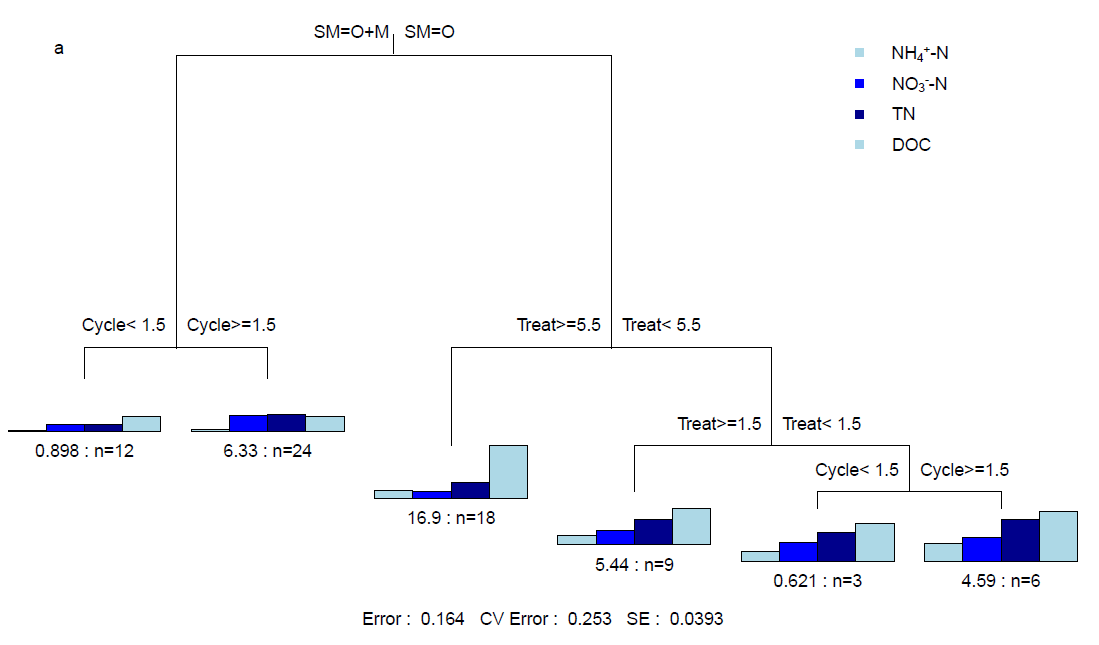

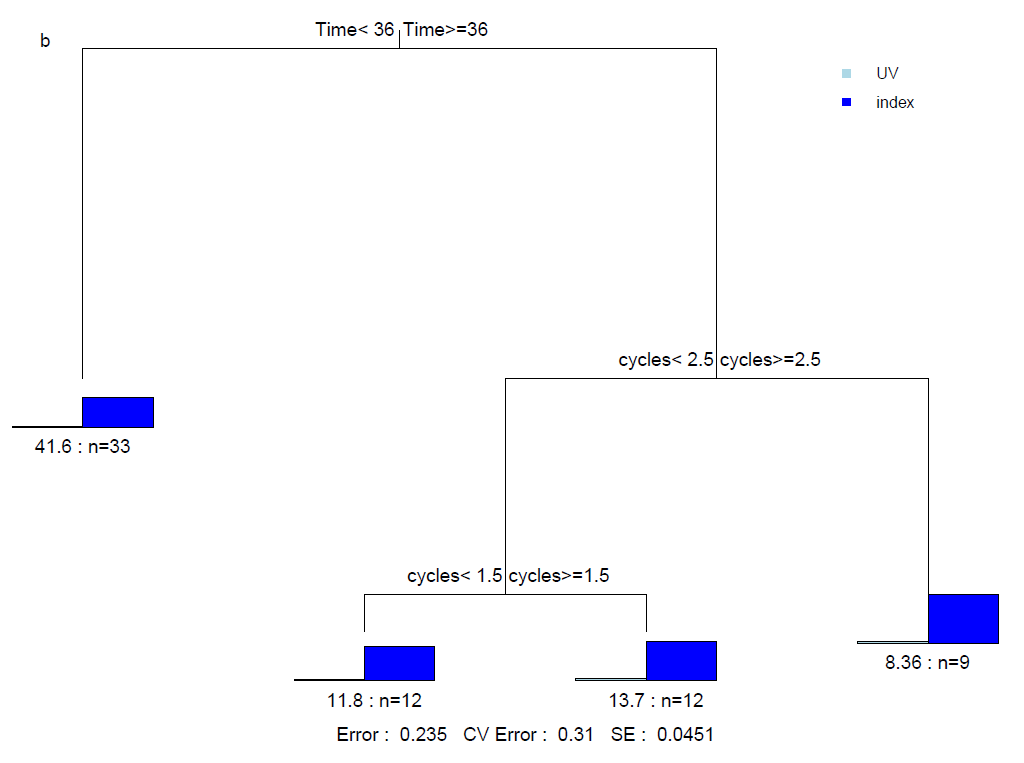


**Figure S6** Multiple Regression Tree analyzes of the impact of freeze-thaw pattern to soil properties in Paper 4. a: FTN: 4.05%; FTF: 16.76%; SM: 60.63%; b: FTN: 33.19%; Time: 43.32%.

**Table S9** Multivariate variance analysis of the effect of freeze-thaw pattern on soil properties in Paper 5

|  |  | nematodes | |  | MB | |  | Basal respiration | |  | NH_4_^+^-N | |  | CO_2_ evolution | |
| --- | --- | --- | --- | --- | --- | --- | --- | --- | --- | --- | --- | --- | --- | --- | --- |
|  | Df | *F* | *p* |  | *F* | *p* |  | *F* | *p* |  | *F* | *p* |  | *F* | *p* |
| FTN | 1 | 406.435 | <0.001*** |  | 0.307 | 0.584 |  | 7.529 | 0.011* |  | 863.285 | <0.001*** |  | 3089.90 | <0.001*** |
| FTF | 1 | 52.614 | <0.001*** |  | 2.309 | 0.121 |  | 2.715 | 0.087 |  | 145.426 | <0.001*** |  | 331.41 | <0.001*** |
| D | 2 | 103.406 | <0.001*** |  | 19.803 | <0.001*** |  | 134.728 | <0.001*** |  | 3.938 | 0.059 |  | 128.93 | <0.001*** |
| D*FTN | 1 | 82.046 | <0.001*** |  | 2.233 | 0.129 |  | 3.590 | 0.043 |  | 235.570 | <0.001*** |  | 502.67 | <0.001*** |
| FTF*D | 2 | 48.020 | <0.001*** |  | 1.638 | 0.213 |  | 2.661 | 0.116 |  | 7.554 | 0.011* |  | 52.71 | <0.001*** |
| FTF*FTN | 2 | 7.828 | 0.002** |  | 3.076 | 0.065 |  | 1.594 | 0.224 |  | 3.471 | 0.047* |  | 13.76 | <0.001*** |
| FTF*D*FTN | 2 | 12.281 | <0.001*** |  | 3.201 | 0.059 |  | 1.336 | 0.282 |  | 5.043 | 0.015* |  | 16.76 | <0.001*** |

D: Decomposer community complexity.


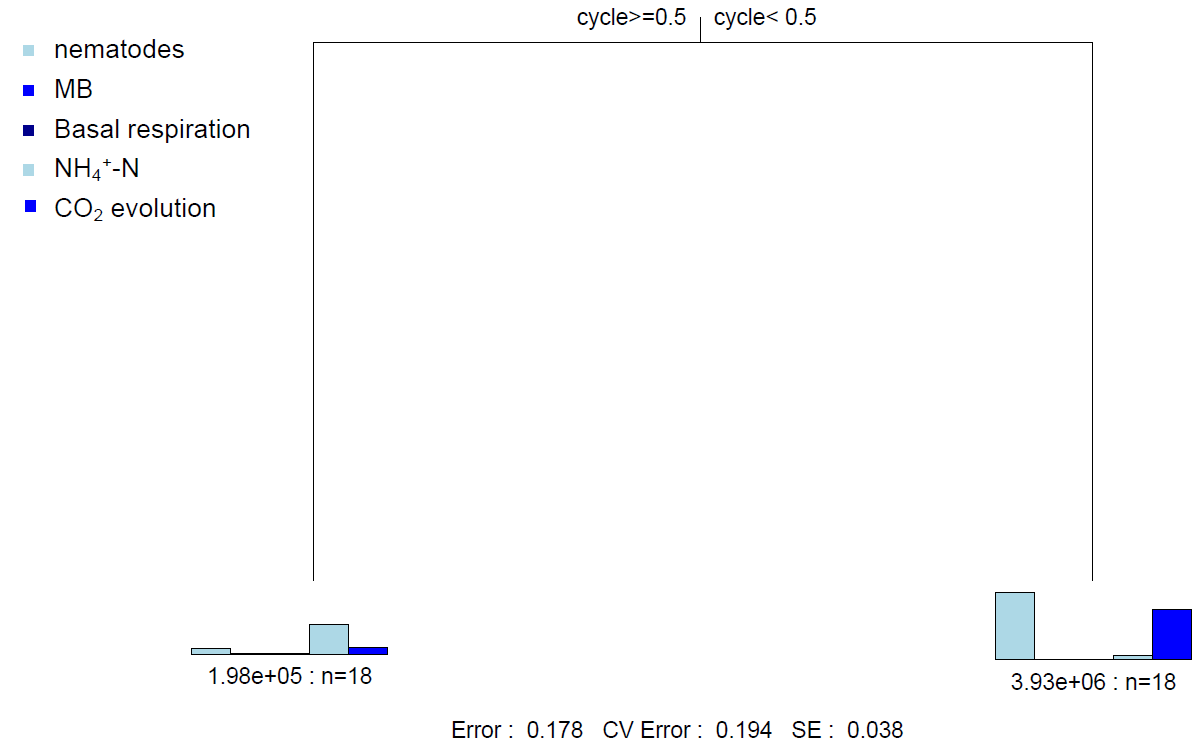


**Figure S7** Multiple Regression Tree analyzes of the impact of freeze-thaw pattern to soil properties in Paper 5. FTN: 82.24%.

**Table S10** Multivariate variance analysis of the effect of freeze-thaw pattern on root properties in Paper 6

|  |  |  | Root damage | |  | REL | |
| --- | --- | --- | --- | --- | --- | --- | --- |
|  |  | Df | *F* | *p* |  | *F* | *p* |
| Holcus lanatus | FTF | 1 | 199.994 | 0.424 |  | 50.66 | <0.001*** |
|  | FTN | 1 | 1208.577 | 0.219 |  | 18.12 | <0.001*** |
|  | FTF*FTN | 23 | - | - |  | 20.33 | <0.001*** |
| Calluna vulgaris adult | FTF | 1 | 2.126 | 0.179 |  | 6.22 | 0.020* |
|  | FTN | 1 | 0.265 | 0.619 |  | 4.014 | 0.057 |
|  | FTF*FTN | 23 | - | - |  | 7.309 | 0.013* |
| Calluna vulgaris juvenil | FTF | 2 | 0.081 | 0.782 |  | - | - |
|  | FTN | 1 | 5.835 | 0.039* |  | - | - |
|  | FTF*FTN | 2 | - | - |  | - | - |


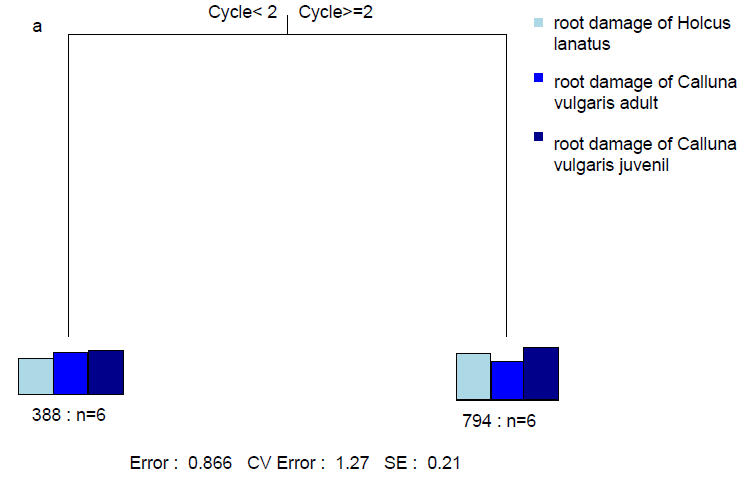

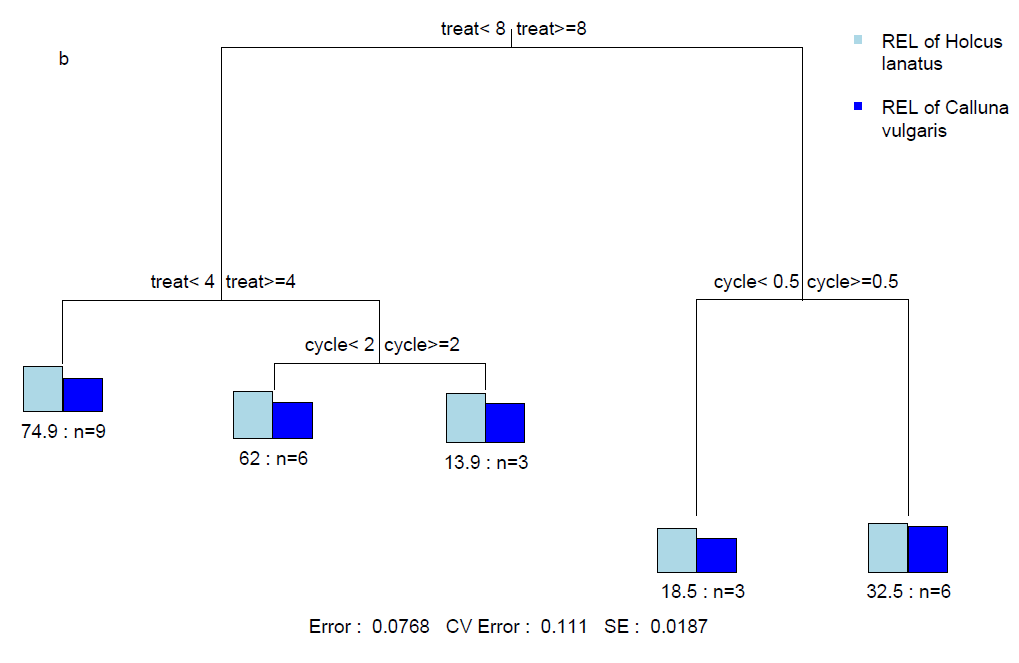


**Figure S8** Multiple Regression Tree analyzes of the impact of freeze-thaw pattern to soil properties in Paper 6. a: FTN: 13.36%; b: FTN: 43.69%; FTF: 48.63%.

**Reference**

Hentschel K, Borken W and Matzner E (2008). Repeated freeze–thaw events affect leaching losses of nitrogen and dissolved organic matter in a forest soil. *J Plant Nutr Soil Sc* 171, 699-706. doi: 10.1002/jpln.200700154

Kreyling J, Peršoh D, Werner S, Benzenberg M and Wöllecke J (2012). Short-term impacts of soil freeze-thaw cycles on roots and root-associated fungi of Holcus lanatusand Calluna vulgaris. *Plant Soil* 353, 19-31. doi: 10.1007/s11104-011-0970-0

Sulkava P and Huhta V (2003). Effects of hard frost and freeze-thaw cycles on decomposer communities and N mineralisation in boreal forest soil. *Appl Soil Ecol* 22, 225-239. doi: 10.1016/s0929-1393(02)00155-5

Wang J, Song C, Hou A, Miao Y, Yang G and Zhang J (2015). Effects of freezing–thawing cycle on peatland active organic carbon fractions and enzyme activities in the Da Xing’anling Mountains, Northeast China. *Environ Earth Sci* 72, 1853-1860. doi: 10.1007/s11104-014-2263-x

Wang W, Shu X, Zhang Q and Guénon R (2015). Effects of freeze–thaw cycles on the soil nutrient balances, infiltration, and stability of cyanobacterial soil crusts in northern China. *Plant Soil* 386, 263-272. doi: 10.1007/s11104-014-2263-x

Zheng Q, Hu Y, Zhang S, Noll L, Böckle T, Dietrich M*, et al.* (2019). Soil multifunctionality is affected by the soil environment and by microbial community composition and diversity. *Soil Biol Biochem* 136, 107521. doi: 10.1016/j.soilbio.2019.107521

Zhou W, Chen H, Zhou L, Lewis BJ, Ye Y, Tian J*, et al.* Effect of freezing-thawing on nitrogen mineralization in vegetation soils of four landscape zones of Changbai Mountain. *Ann Forest Sci* 68, 943-951. doi: 10.1007/s13595-011-0100-4
